# The relationship between low maternal serum vitamin D levels and glycemic control in gestational diabetes assessed by HbA1c levels: an observational cross-sectional study

**DOI:** 10.1186/1471-2393-14-362

**Published:** 2014-10-13

**Authors:** Ahmed El Lithy, Rana M Abdella, Yahia M El-Faissal, Ahmed M Sayed, Rasha M Abdel Samie

**Affiliations:** Obstetrics and Gynecology Department, Cairo University, Cairo, Egypt; General Medicine Departments, Cairo University, Cairo, Egypt

**Keywords:** Vitamin D, Gestational diabetes mellitus, Glycemic control, Insulin sensitivity

## Abstract

**Background:**

A great association between vitamin D deficiency and type 2 diabetes mellitus has been suggested in literature. During pregnancy, this deficiency is even more critical. It appears that vitamin D insufficiency during pregnancy may be associated with maternal hazards. The aim of this study was to assess the relation between the levels of 25-hydroxy-cholecalciferol (vitamin D), and the glycemic control in pregnant women.

**Methods:**

An observational cross-section study including 160 pregnant women between 20-40 years in age, in their third trimester, divided into two equal groups. First group consisted of 80 women with established diagnosis of gestational diabetes and the second group with proved normal blood glucose levels. We assessed vitamin D in serum, fasting blood glucose, serum insulin and glycosylated hemoglobin (HbA1c) levels and we depicted the insulin sensitivity using the Quantitative insulin sensitivity check index (Quicki). The results were collected and statistically correlated.

**Results:**

The mean vitamin D levels were 46.61 ± 6.087 and 47.25 ± 10.181in controls and women with gestational diabetes mellitus (GDM) respectively. The fasting insulin levels were significantly higher in the group with GDM with a mean of 18.51 ± 6.44 compared to 8.95 ± 2.52 in the control group.

The correlation coefficient (r) between HbA1c levels and Vitamin D level was -0.492 with a P value <0.05. Similar associations were also found with the fasting blood sugar levels (r = - 0.386) and with Quicki values (r = -0.250). Vitamin D levels correlated significantly with the fasting blood glucose, the fasting serum insulin and the HbA1c levels, the P value in all these correlations were <0.05. The P value with Quicki results was 0.064.

**Conclusions:**

There is a statistically significant negative correlation between the glycemic control and vitamin D levels in serum in the whole study population. The effect of adequate vitamin D replacement on glycemic control was not studied in our work correlation. We suggest larger scale studies addressing this issue.

## Background

Vitamin D acting on its own receptors can produce a number of desired biological effects via different mechanisms and, therefore, contributes to the improvement of human health [1]. After absorption, vitamin D is bound to the binding protein and carried to the liver via the bloodstream. From there it begins two hydroxylation processes; beginning in the liver it is transformed into 25(OH) vitamin D (calcidiol), and finally, in the kidneys, where it is transformed into 1,25 dihydroxy-vitamin D (calcitriol), which is the biologically active form of vitamin D. The levels of serum active vitamin D; 1,25dihydroxy-cholecalciferol (1,25(OH)_2_ 2D) increase during pregnancy as depicted by several studies, the general conclusion linked this serum rise to increased renal hydroxylation of 25-hydroxy-cholecalciferol (25(OH) D) to 1,25(OH)_2_ 2D under stimulation of pregnancy [2].

There’s a great association between vitamin D deficiency and type 2 diabetes mellitus [3]. In addition, some papers reported a prevalence of inadequate 25(OH)D levels in 41% of the women with GDM, and they consequently proposed routine 25(OH) D testing of all pregnant women when screening for GDM or earlier, and treatment of women who are found to be deficient [4].

This proposition was the rationale behind this study; we found a need to study the status of vitamin D deficiency amongst Egyptian females and its relation to the glycemic control in pregnant women with GDM, and we aimed at producing recommendations concerning this issue to be implemented in the current Egyptian antenatal care program.

We included in this study the assessment of the glycated haemoglobin (HbA1c). The glycation of haemoglobin was first used 25 years ago to assess the level of glycaemia in subjects with diabetes [5]. Since then, assay of HbA1c has become the ‘gold standard’ by which the effectiveness of glycemic control is judged in clinical practice [6].

### Aim of work

This study investigated the relation between vitamin D3 level and the glycemic control assessed by HbA1c, blood glucose levels, Quicki and insulin in serum in gestational diabetes mellitus.

## Methods

This cross-sectional observational study was conducted at Cairo University hospital; Kasr al-Aini, the obstetrics and gynecology department, during the period between June 2012 and June 2013. It was approved by the Scientific & Ethical Committee of the Obstetrics and Gynecology Department of Cairo University. We approached the pregnant women, in the third trimester, attending the antenatal care clinic of Cairo university hospital during this period, the target age group was between 20-40 years. We first enrolled 80 pregnant women with established diagnosis of gestational diabetes. Those women were diagnosed as having GDM as depicted by estimating the fasting and 2-hours post-prandial blood glucose levels in previous antenatal care visit; they were already receiving treatment for their condition, through a collaborative approach, involving the Diabetes Mellitus outpatient clinic and the internal medicine department of Cairo University. We then included a second group; consisting of randomly selected 80 pregnant women, those women had normal fasting and 2-hours post-prandial blood glucose levels blood glucose levels, during their routine antenatal care visits. The need to include a control group emerged when we were faced by the high incidence of vitamin D deficiency among our included cases of the first group. A question was raised; is this deficiency caused by impaired glucose tolerance, or it is a common finding in the population study. We included an equal number to the previously enrolled pregnant diabetic women, in order to obtain statistical equality. Written Informed consents were obtained from each participant prior to enrollment; the consent form was written in Arabic language and followed the world health organization guidelines (WHO). All consent forms were signed by one of the investigators, and were collected and filed. Patients giving history of type 1 or 2 diabetes mellitus antedating pregnancy were excluded, as well as any medical disorders (such as hypertension or cardiac disease) or medical complications related to pregnancy. All the patients included were subjected to detailed history taking with special focus on maternal age, parity, gestational age at diagnosis of gestational diabetes, previous history or family history of diabetes, history of gestational diabetes in previous pregnancies. Data concerning insulin regimens, insulin types and doses (if diagnosed of having gestational diabetes) were also noted. Body Mass Index (BMI) was calculated for all subjects as follows; weight in kilograms divided by their height in meters squared (18.5 or less: underweight, 18.5-24.9 is normal, 25-29.9: overweight, 30-39.9: Obese and 40 or greater is extremely obese). The BMI in the control group was matched to that in the GDM group for sakes of statistical adjustment. Laboratory investigation included Vitamin D3 level measured by radio immunoassay. Vitamin D deficiency was defined conservatively as <25 ɳmol/L, insufficiency as 25–50 ɳmol/L and sufficiency as >50 ɳmol/L. HbA1c was measured by radioimmunoassay to assess the glycemic control. The normal level for glycosylated hemoglobin is less than 7%. Diabetics rarely achieve such levels, but tight control aims to come close to it. Levels above 9% show poor control, and levels above 12% show very poor control. Fasting serum insulin (in mIU/L), fasting blood glucose and 2 hours postprandial, interpreted in mg/dL were included within the laboratory investigations. The insulin sensitivity as a reflection of insulin resistance was measured using the Quantitative insulin sensitivity check index (Quicki). This index correlates well with glucose clamp studies (r =0.78), and it has the advantage of that it can be obtained from a fasting blood sample, hence it is the preferred method for several types of clinical research [7]. It is calculated as {1/( log fasting insulin + log fasting blood sugar). Blood samples were collected from pregnant women with gestational diabetes during pregnancy during routine antenatal care visits and were analyzed in Cairo University Clinical Pathology Department’s laboratories. All results were statistically analyzed and tabulated.

### Statistical analysis

Data were statistically described in terms of mean ± standard deviation (±SD), median and range, or frequencies (number of cases) and percentages when appropriate. Comparison of numerical variables between the study groups was done using Student t test for independent samples. For comparing categorical data, Chi square test was performed. Exact test was used instead when the expected frequency is less than 5. P values less than 0.05 was considered statistically significant. All statistical calculations were done using computer programs SPSS (Statistical Package for the Social Science; SPSS Inc., Chicago, IL, USA) version 15 for Microsoft Windows.

## Results

One hundred and sixty pregnant women, in their third trimester, consented to participate in this study. They were all comparable regarding the maternal age, pre-pregnancy body mass index (BMI), and parity as shown in Table 1. The fasting plasma glucose level was significantly lower in controls compared to those with gestational diabetes. 36.25% of cases with gestational diabetes gave history of GDM in previous pregnancies which was also statistically significant (p-value = 0.0001), also family history of type 2 DM seemed more likely in this same group. The majority of the controls and women with GDM suffered from Vitamin D insufficiency rather than deficiency where the mean 25 OHD levels were 46.61 ± 6.087 and 47.25 ± 10.181 respectively. The fasting insulin levels were significantly higher in the group with GDM with a mean of 18.51 ± 6.44 compared to 8.95 ± 2.52 in the control group.

**Table 1:** **Clinical and biochemical demographic data of the studied groups**

	Controls (n = 80)	Cases with GDM (n = 80)	P-value
**Maternal age in years**	24.40 +/- 3.77	25.38 +/- 3.83	0.107 (NS)
**Pre-pregnancy BMI (Kg/m2)**	25.31 +/- 2.39	26.46 +/- 2.95	0.665 (NS)
**Parity**	1.79 +/- 1.06	1.81 +/- 1.02	0.88 (NS)
**Previous history of GDM**	5 (6.25%)	29 (36.25%)	0.0001(S)
**Family history of type 2 DM**	9 (11.25%)	36 (45%)	0.0001(S)
**Fasting blood sugar mg/dl**	77.05 +/- 7.15	94.05 +/- 14.33	0.000 (S)
**25OHD (nmol/L)**	46.61 +/- 6.08	47.25 +/- 10.18	0.632 (NS)
**HbA1C**	4.47 +/- 0.54	4.25 +/- 1.09	0.117 (NS)
**Fasting insulin**	8.95 +/- 2.52	18.51 +/- 6.44	0.000 (S)
**Quicki**	0.3518 +/- 0.1784	0.4848 +/- 0.0195	0.000 (S)

25OHD: 25-hydroxyvitamin D, BMI: body mass index, DM: diabetes mellitus, GDM: gestational diabetes mellitus, NS: not significant, S:significant, OGTT: oral glucose tolerance test, PGL:plasma glucose.

A significant inverse correlation was found between the HbA1C levels and Vitamin D levels (correlation coefficient r = -0.492, P value <0.05 ), where the higher the levels of Vitamin D, the lower the HbA1c levels indicating a good glycemic control in women with gestational diabetes. An inverse association was also found with the fasting blood sugar levels which was statistically significant (the correlation coefficient r = - 0.386), as well as with Quicki values (r = - 0.250) (Table 2).

**Table 2:** **The correlation between H** ***bA1C*** **and different maternal biochemical variables in women with gestational diabetes**

Variables	Correlation coefficient (r value)	P-value	Significance
**Fasting blood sugar**	- 0.386	0.000	S
**Fasting insulin levels**	0.332	0.000	S
**Vitamin D levels**	-0.492	0.000	S
**QUICKI equation**	-0.250	0.001	S

**Figure 1:**
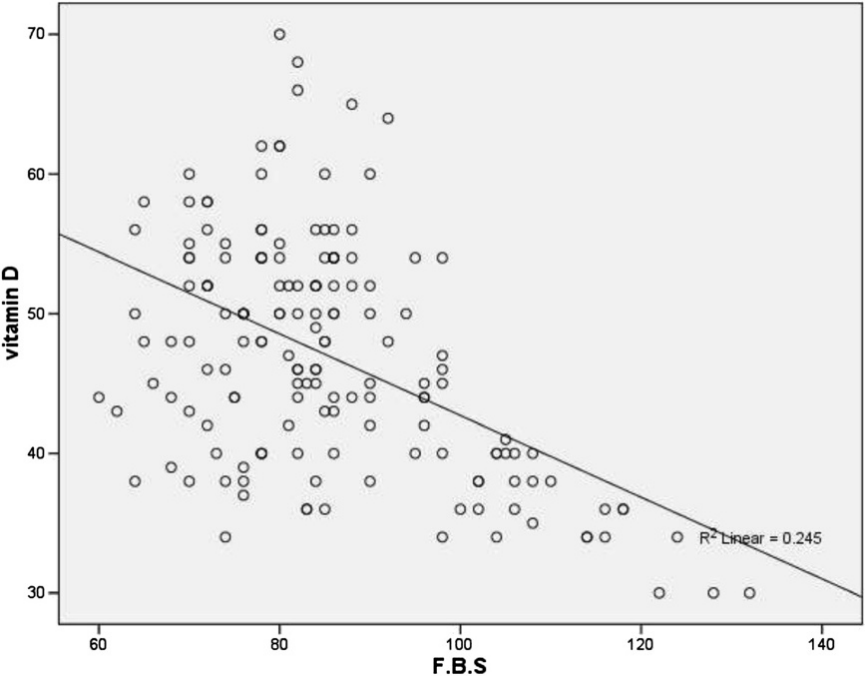
**The scatter plot shows negative linear correlation between fasting blood glucose and Vitamin D level (r = - 0.245).**

As regards the Vitamin D levels, Table 3 shows that they negatively correlated with the fasting blood glucose levels (Figure 1), the fasting serum insulin levels and the HbA1c levels, in the whole study population including both groups; women with GDM and controls. The P value in all these correlations were <0.05. The Quicki values, however, did not significantly correlate with the Vitamin D levels, the P value being 0.064.

**Table 3:** **The correlation between the Vitamin D levels and the various maternal biochemical variables in women included in the study**

Variable	Correlation coefficient (r value)	P-value	Significance
**Fasting blood sugar**	-0.245	0.000	S
**Fasting insulin levels**	-0.357	0.000	S
**HbA1C levels**	-0.492	0.000	S
**Quicki equation**	0.147	0.064	NS

## Discussion

Vitamin D plays a great role in bone metabolism and mineral homeostasis. Relevant data identify roles for the active form of vitamin D (1,25(OH)_2_D3) in many biological processes including regulation of cellular growth, differentiation and metabolic modulations [8]. Vitamin D insufficiency has a known effect on bone density, neonatal vitamin D and calcium status, and childhood rickets [9]. In several studies, the relation between low vitamin D levels, insulin resistance and impaired insulin secretion was clearly demonstrated [10]. Moreover, specific receptors for 1,25(OH)_2_D3 were detected in pancreatic ß cells, denoting a probable effect of vitamin D on the insulin secretion process [11]. Obesity is another significant risk factor for 25OHD deficiency [12, 13]. An analysis of the National Health and Nutrition Examination Survey (NHANES) for the years 2003/2004 data also demonstrated that 25 (OH) D deficiencies were highly prevalent in overweight and obese American subjects [14].

In our study we tried to find a correlation between glycemic control and vitamin D deficiency in pregnant women known to have gestational diabetes, diagnosed at previous visits, and we included normal pregnant population as a control. Vitamin D deficiency was the salient feature in both groups, with the mean serum vitamin D levels close in both groups. This could be explained by the fact that the great majority of the pregnant women, included in our study, belonged to poor economic standards which may affect a good supplementation of vitamin D; they have poor housing conditions, poor exposure to sun, insufficient food intake which might be referred to as vegetarian in other papers, repeated non spaced pregnancies and lack of medical service to provide good vitamin supplementation. Another important factor is that, in spite of the sunny climate in Egypt, all the included women were veiled, which hinder the passage of sunrays to the skin, and subsequently diminishing trans-cutaneous vitamin D absorption. We had a negative linear correlation between fasting blood glucose levels and vitamin D3. Our findings also support an independent inverse association between 25(OH)D and HbA1c in women with GDM, showing a potential interaction between 25(OH)D and blood glucose control in pregnancy. The higher levels of HbA1c, indicating poor glycemic control, were found in women with lower levels of 25(OH)D and this inverse relation was found to be statistically significant (r = - 0.492).

The possible explanation for such relationship, between Vitamin D deficiency and the impaired glycemic control, found in our study could be made attributed to the defect in the important role that Vitamin D plays in glucose homeostasis, and via different mechanisms. The mechanism of action of vitamin D in type 2 diabetes is thought to be mediated not only through regulation of plasma calcium levels, which regulate insulin synthesis and secretion, but it also improves insulin sensitivity of the target cells (liver, skeletal muscle, and adipose tissue). Additionally, Vitamin D enhances and improves β-cell function and protects them from detrimental immune attacks, directly by its action on β-cells, but also indirectly by acting on different immune cells, including inflammatory macrophages, dendritic cells, and a variety of T cells. Macrophages, dendritic cells, T lymphocytes, and B lymphocytes can synthesize Vitamin D, all contributing to the regulation of local immune responses [15].

This correlation between vitamin D and GDM were evaluated by various studies and the results were controversial [16–19]. Maghbooli et al demonstrated that maternal serum levels of 25(OH)D during 24-28 weeks of pregnancy were significantly lower in women with GDM compared with controls [16]. More recently, Makgoba and his colleagues did not find an association between first trimester maternal serum 25(OH)D levels and subsequent GDM development [17]. On the other way round, Clifton-Bligh demonstrated an inverse association between maternal serum 25(OH)D levels and fasting blood glucose, although the association between 25(OH)D and GDM was not statistically significant [18]. Additionally, a recent study of Indian women failed to demonstrate a relationship between maternal vitamin D status and risk of developing GDM [19]. More recently meta-analysis, published in 2013, linked gestational diabetes with insufficient 25(OH)D levels, it correlated in addition vitamin D deficiency to preeclampsia, preterm birth and small for gestational age (SGA) babies [20]. In 2013, a randomized controlled study from a Turkish group, on 234 women with GDM and 168 controls, came to a more specific conclusion; they found a statistical significance, between glycemic control and vitamin D levels, only in women with severe deficiency of 25(OH)D levels. They also concluded that only this group (with severe vitamin D deficiency) is at a higher risk of GDM [21]. We mostly attribute this disparity in the results and correlations to the population differences, the most influential factor being, from our point of view, the absence or very few numbers of pregnant women with severely deficient 25OHD.

An important limitation of the present study is the small number of patients with severe vitamin D deficiency and the single measurement of 25(OH)D levels in the third trimester of pregnancy. Other confounding factors included the seasonal variation, as the study extended over a whole year, the heterogeneity of dietary habits and the differences in socioeconomic standards between the included pregnant women. We believe however that these factors did not have a major impact on the statistical analysis of this study, considering that most, if not all, of our patients live in the poor socioeconomic areas.

## Conclusions

In our study, there was high prevalence of vitamin D insufficiency and deficiency. Considering this we suggest routine testing of all pregnant women, and treatment of women who are found to be vitamin D deficient. We do not know whether adequate vitamin D replacement may contribute to prevention of GDM later in pregnancy, as our study suggests a significant inverse correlation between vitamin D status and glycemic control in all the pregnant women included. We recommend larger scale studies addressing this issue, with a special concern to the effectiveness of adequate replacement of vitamin D in preventing GDM.

## Abbreviations

GDM: Gestational diabetes mellitus
BMI: Body mass index
Quicki: Quantitative insulin sensitivity check index
NHANES: National health and nutrition examination survey
S: Significant
NS: Not significant.
